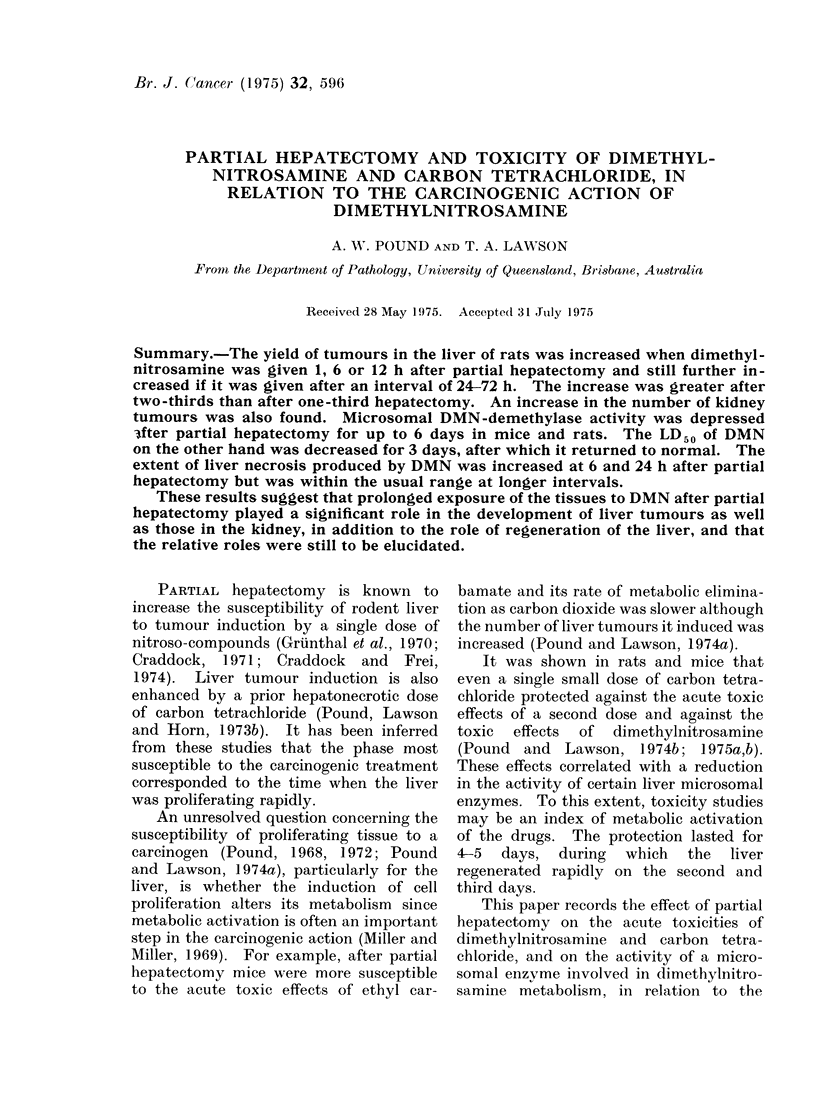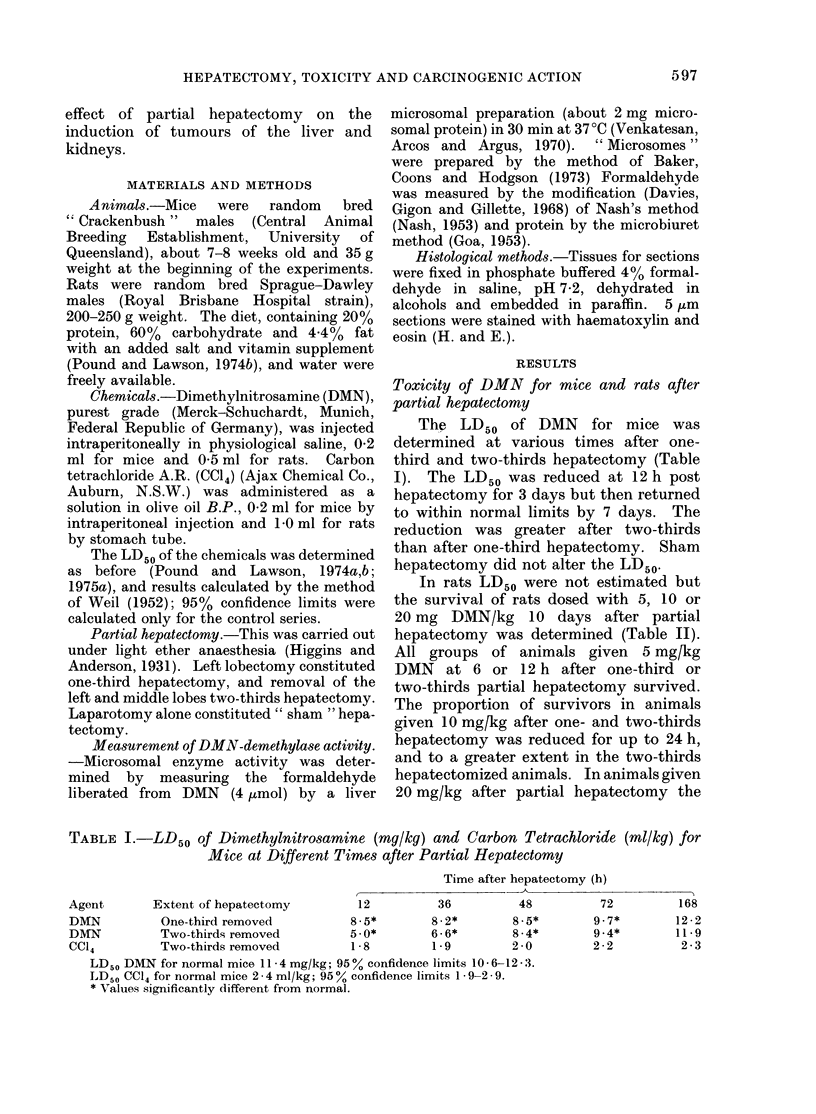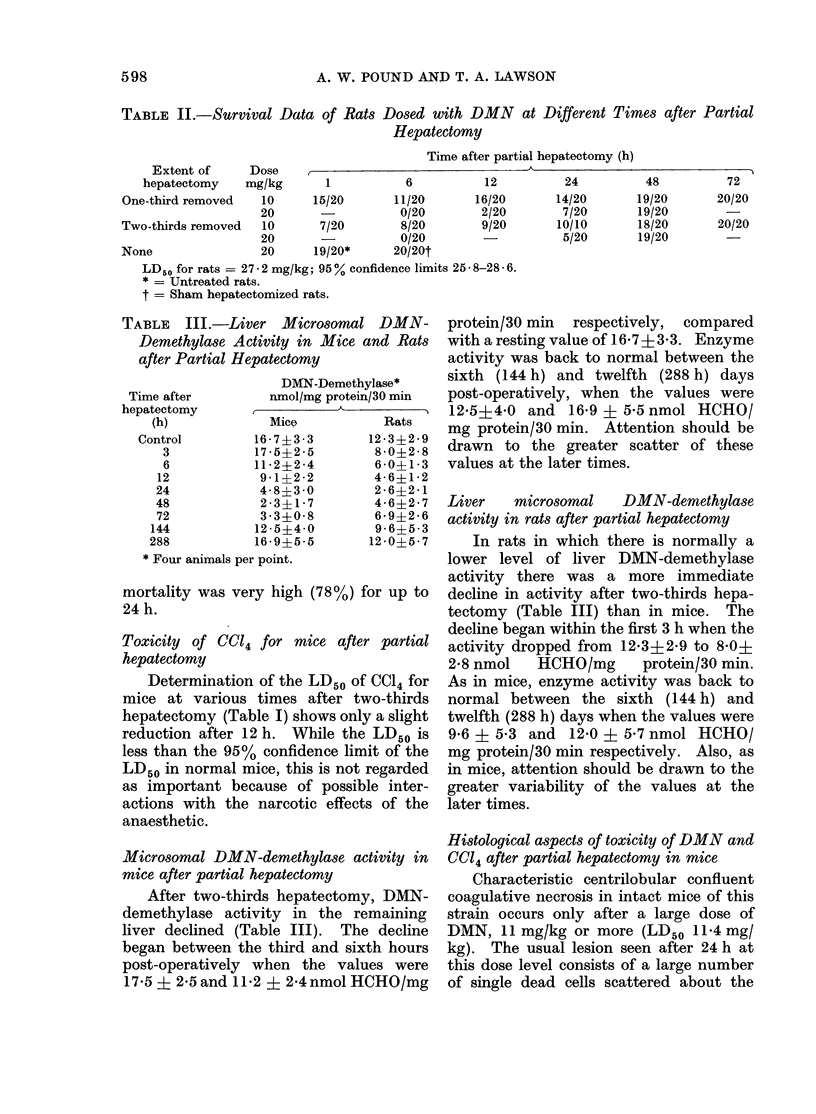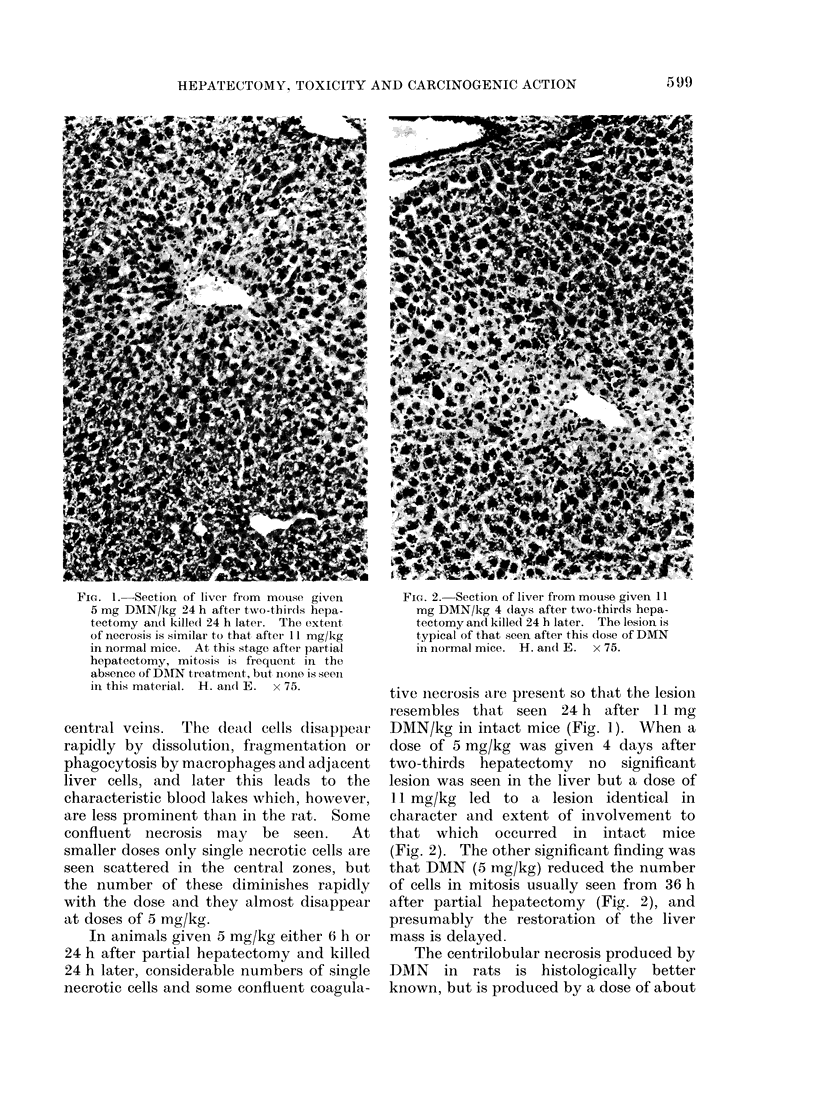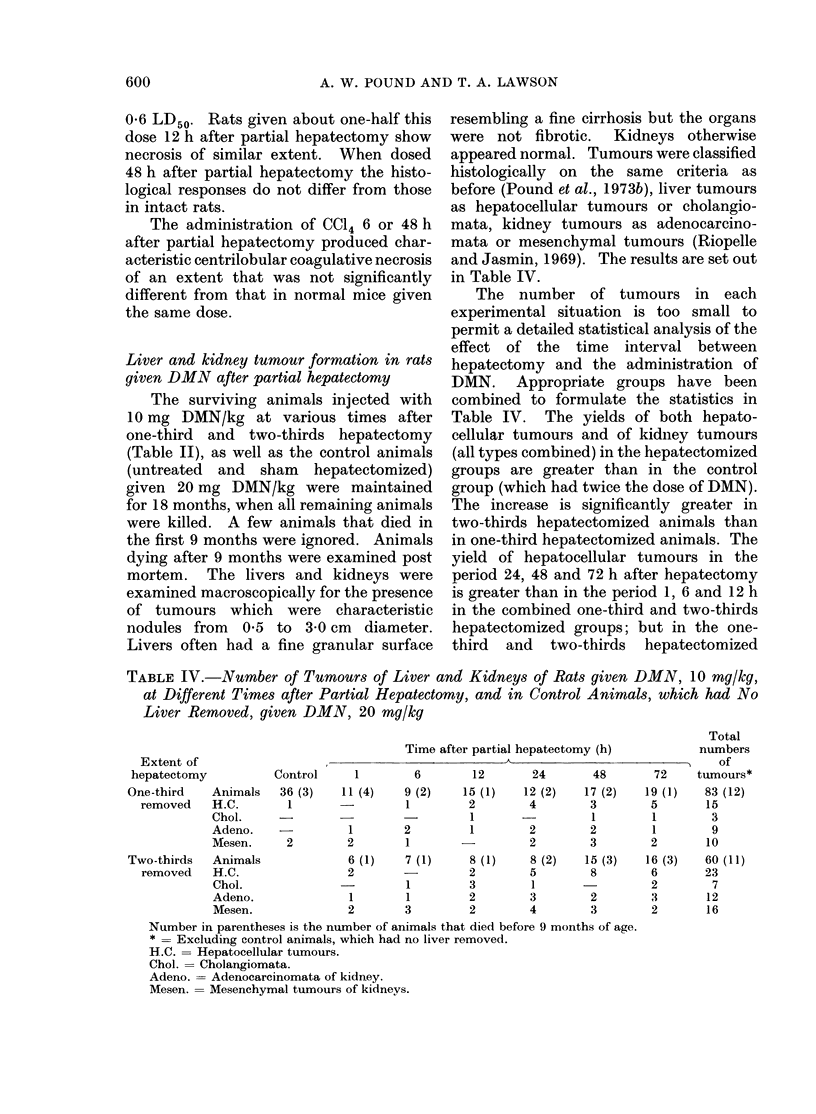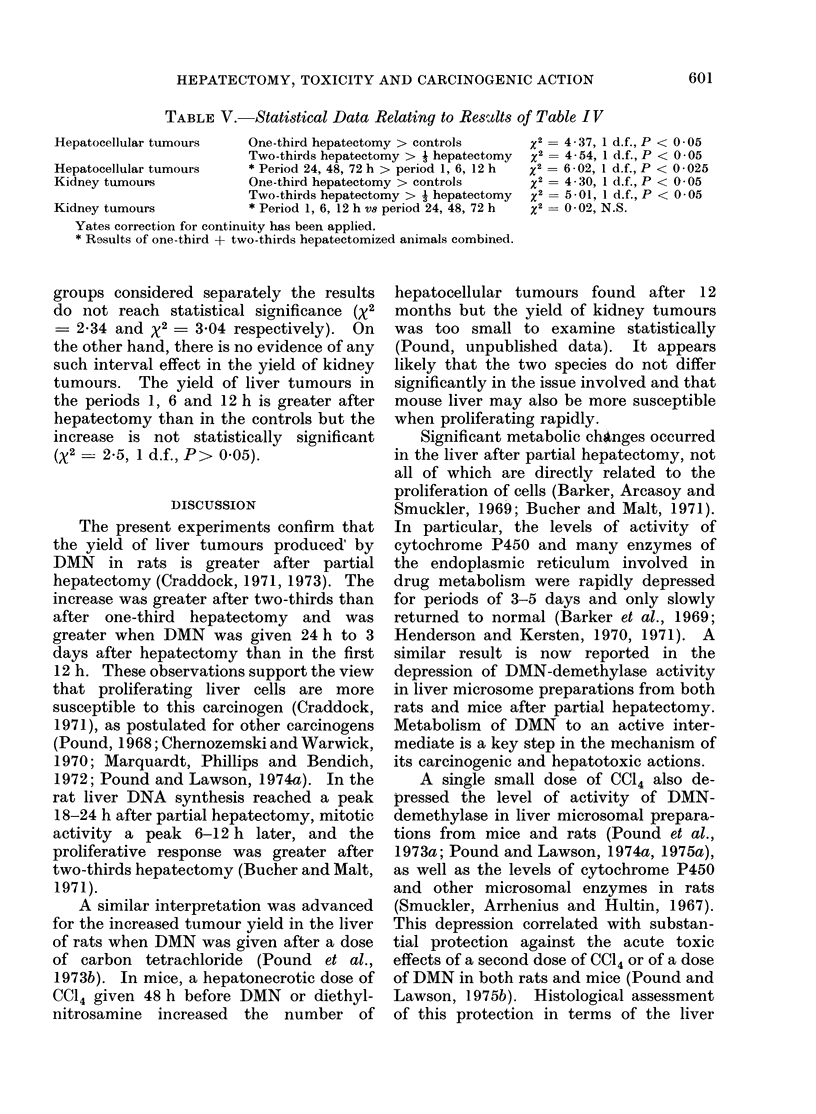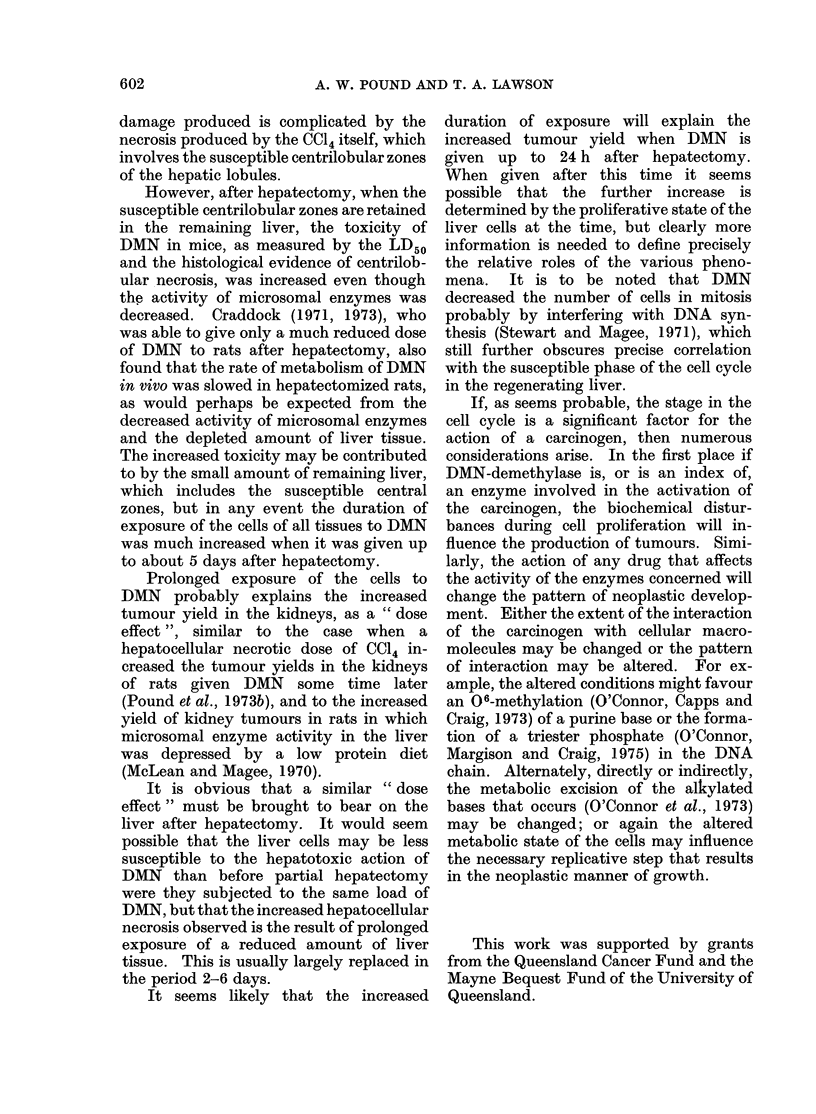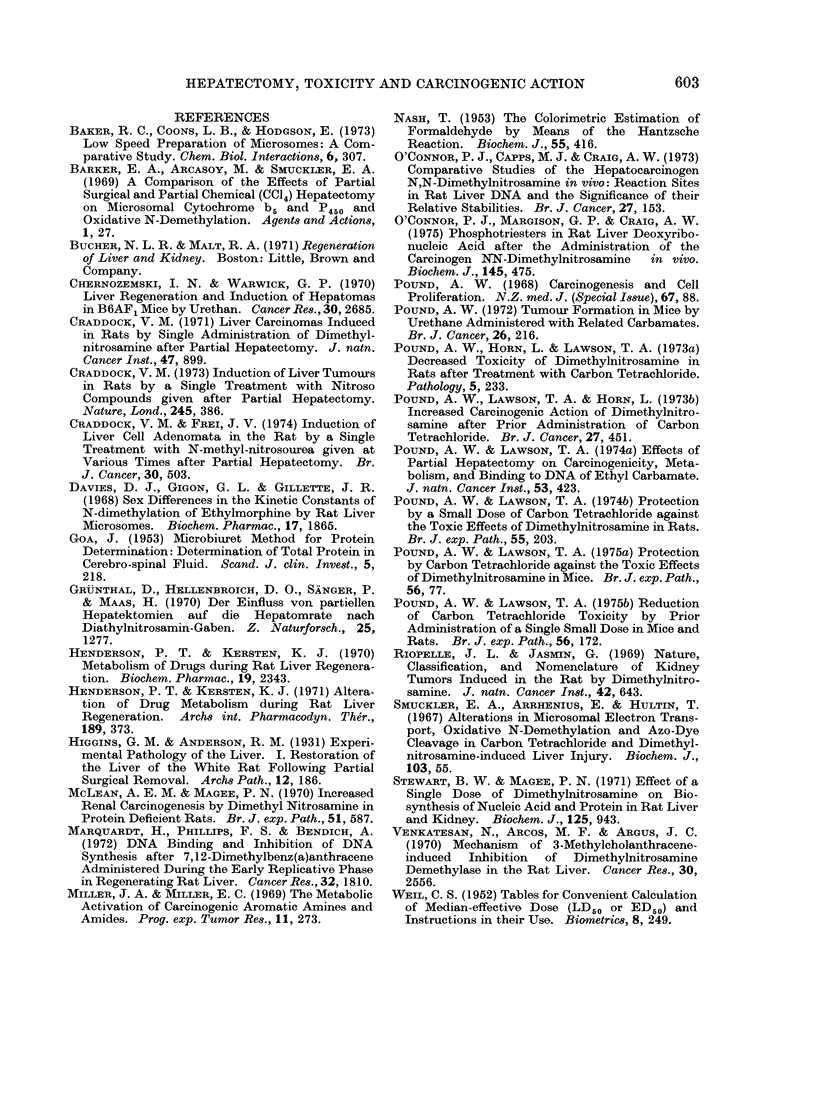# Partial hepatectomy and toxicity of dimethyl-nitrosamine and carbon tetrachloride, in relation to the carcinogenic action of dimethylnitrosamine.

**DOI:** 10.1038/bjc.1975.266

**Published:** 1975-11

**Authors:** A. W. Pound, T. A. Lawson

## Abstract

**Images:**


Br. J. Cancer (1975) 32, 596

### PARTIAL HEPATECTOMY AND TOXICITY OF DIMETHYLNITROSAMINE AND CARBON TETRACHLORIDE, IN RELATION TO THE CARCINOGENIC ACTION OF DIMETHYLNITROSAMINE

A. W. POUND AND T. A. LAWSON

*From the Department of Pathology, University of Queensland, Brisbane, Australia*

Received 28 May 1975. Accepted 31 July 1975

**Summary.—The yield of tumours in the liver of rats was increased when dimethylnitrosamine was given 1, 6 or 12 h after partial hepatectomy and still further increased if it was given after an interval of 24–72 h. The increase was greater after two-thirds than after one-third hepatectomy. An increase in the number of kidney tumours was also found. Microsomal DMN-demethylase activity was depressed after partial hepatectomy for up to 6 days in mice and rats. The $LD_{50}$ of DMN on the other hand was decreased for 3 days, after which it returned to normal. The extent of liver necrosis produced by DMN was increased at 6 and 24 h after partial hepatectomy but was within the usual range at longer intervals.**

**These results suggest that prolonged exposure of the tissues to DMN after partial hepatectomy played a significant role in the development of liver tumours as well as those in the kidney, in addition to the role of regeneration of the liver, and that the relative roles were still to be elucidated.**

PARTIAL hepatectomy is known to increase the susceptibility of rodent liver to tumour induction by a single dose of nitroso-compounds (Grünthal *et al.*, 1970; Craddock, 1971; Craddock and Frei, 1974). Liver tumour induction is also enhanced by a prior hepatonecrotic dose of carbon tetrachloride (Pound, Lawson and Horn, 1973*b*). It has been inferred from these studies that the phase most susceptible to the carcinogenic treatment corresponded to the time when the liver was proliferating rapidly.

An unresolved question concerning the susceptibility of proliferating tissue to a carcinogen (Pound, 1968, 1972; Pound and Lawson, 1974*a*), particularly for the liver, is whether the induction of cell proliferation alters its metabolism since metabolic activation is often an important step in the carcinogenic action (Miller and Miller, 1969). For example, after partial hepatectomy mice were more susceptible to the acute toxic effects of ethyl carbamate and its rate of metabolic elimination as carbon dioxide was slower although the number of liver tumours it induced was increased (Pound and Lawson, 1974*a*).

It was shown in rats and mice that even a single small dose of carbon tetrachloride protected against the acute toxic effects of a second dose and against the toxic effects of dimethylnitrosamine (Pound and Lawson, 1974*b*; 1975*a,b*). These effects correlated with a reduction in the activity of certain liver microsomal enzymes. To this extent, toxicity studies may be an index of metabolic activation of the drugs. The protection lasted for 4–5 days, during which the liver regenerated rapidly on the second and third days.

This paper records the effect of partial hepatectomy on the acute toxicities of dimethylnitrosamine and carbon tetrachloride, and on the activity of a microsomal enzyme involved in dimethylnitrosamine metabolism, in relation to the effect of partial hepatectomy on the induction of tumours of the liver and kidneys.

### MATERIALS AND METHODS

*Animals*.—Mice were random bred " Crackenbush " males (Central Animal Breeding Establishment, University of Queensland), about 7–8 weeks old and 35 g weight at the beginning of the experiments. Rats were random bred Sprague–Dawley males (Royal Brisbane Hospital strain), 200–250 g weight. The diet, containing 20% protein, 60% carbohydrate and 4·4% fat with an added salt and vitamin supplement (Pound and Lawson, 1974*b*), and water were freely available.

*Chemicals*.—Dimethylnitrosamine (DMN), purest grade (Merck–Schuchardt, Munich, Federal Republic of Germany), was injected intraperitoneally in physiological saline, 0·2 ml for mice and 0·5 ml for rats. Carbon tetrachloride A.R. ($CCl_4$) (Ajax Chemical Co., Auburn, N.S.W.) was administered as a solution in olive oil *B.P.*, 0·2 ml for mice by intraperitoneal injection and 1·0 ml for rats by stomach tube.

The $LD_{50}$ of the chemicals was determined as before (Pound and Lawson, 1974*a*,*b*; 1975*a*), and results calculated by the method of Weil (1952); 95% confidence limits were calculated only for the control series.

*Partial hepatectomy*.—This was carried out under light ether anaesthesia (Higgins and Anderson, 1931). Left lobectomy constituted one-third hepatectomy, and removal of the left and middle lobes two-thirds hepatectomy. Laparotomy alone constituted " sham " hepatectomy.

*Measurement of DMN-demethylase activity*.—Microsomal enzyme activity was determined by measuring the formaldehyde liberated from DMN (4 μmol) by a liver microsomal preparation (about 2 mg microsomal protein) in 30 min at 37 °C (Venkatesan, Arcos and Argus, 1970). " Microsomes " were prepared by the method of Baker, Coons and Hodgson (1973) Formaldehyde was measured by the modification (Davies, Gigon and Gillette, 1968) of Nash's method (Nash, 1953) and protein by the microbiuret method (Goa, 1953).

*Histological methods*.—Tissues for sections were fixed in phosphate buffered 4% formaldehyde in saline, pH 7·2, dehydrated in alcohols and embedded in paraffin. 5 μm sections were stained with haematoxylin and eosin (H. and E.).

### RESULTS

### *Toxicity of DMN for mice and rats after partial hepatectomy*

The $LD_{50}$ of DMN for mice was determined at various times after one-third and two-thirds hepatectomy (Table I). The $LD_{50}$ was reduced at 12 h post hepatectomy for 3 days but then returned to within normal limits by 7 days. The reduction was greater after two-thirds than after one-third hepatectomy. Sham hepatectomy did not alter the $LD_{50}$.

In rats $LD_{50}$ were not estimated but the survival of rats dosed with 5, 10 or 20 mg DMN/kg 10 days after partial hepatectomy was determined (Table II). All groups of animals given 5 mg/kg DMN at 6 or 12 h after one-third or two-thirds partial hepatectomy survived. The proportion of survivors in animals given 10 mg/kg after one- and two-thirds hepatectomy was reduced for up to 24 h, and to a greater extent in the two-thirds hepatectomized animals. In animals given 20 mg/kg after partial hepatectomy the

TABLE I.—*$LD_{50}$ of Dimethylnitrosamine (mg/kg) and Carbon Tetrachloride (ml/kg) for Mice at Different Times after Partial Hepatectomy*

| Agent | Extent of hepatectomy | Time after hepatectomy (h) 12 | 36 | 48 | 72 | 168 |
|---|---|---|---|---|---|---|
| DMN | One-third removed | 8·5* | 8·2* | 8·5* | 9·7* | 12·2 |
| DMN | Two-thirds removed | 5·0* | 6·6* | 8·4* | 9·4* | 11·9 |
| $CCl_4$ | Two-thirds removed | 1·8 | 1·9 | 2·0 | 2·2 | 2·3 |

$LD_{50}$ DMN for normal mice 11·4 mg/kg; 95% confidence limits 10·6–12·3.
$LD_{50}$ $CCl_4$ for normal mice 2·4 ml/kg; 95% confidence limits 1·9–2·9.
* Values significantly different from normal.

TABLE II.—*Survival Data of Rats Dosed with DMN at Different Times after Partial Hepatectomy*

| Extent of hepatectomy | Dose mg/kg | Time after partial hepatectomy (h) 1 | 6 | 12 | 24 | 48 | 72 |
|---|---|---|---|---|---|---|---|
| One-third removed | 10 | 15/20 | 11/20 | 16/20 | 14/20 | 19/20 | 20/20 |
| | 20 | — | 0/20 | 2/20 | 7/20 | 19/20 | — |
| Two-thirds removed | 10 | 7/20 | 8/20 | 9/20 | 10/10 | 18/20 | 20/20 |
| | 20 | — | 0/20 | — | 5/20 | 19/20 | — |
| None | 20 | 19/20* | 20/20† | | | | |

$LD_{50}$ for rats = 27·2 mg/kg; 95% confidence limits 25·8–28·6.
* = Untreated rats.
† = Sham hepatectomized rats.

TABLE III.—*Liver Microsomal DMN-Demethylase Activity in Mice and Rats after Partial Hepatectomy*

| Time after hepatectomy (h) | DMN-Demethylase* nmol/mg protein/30 min Mice | Rats |
|---|---|---|
| Control | 16·7±3·3 | 12·3±2·9 |
| 3 | 17·5±2·5 | 8·0±2·8 |
| 6 | 11·2±2·4 | 6·0±1·3 |
| 12 | 9·1±2·2 | 4·6±1·2 |
| 24 | 4·8±3·0 | 2·6±2·1 |
| 48 | 2·3±1·7 | 4·6±2·7 |
| 72 | 3·3±0·8 | 6·9±2·6 |
| 144 | 12·5±4·0 | 9·6±5·3 |
| 288 | 16·9±5·5 | 12·0±5·7 |

* Four animals per point.

mortality was very high (78%) for up to 24 h.

*Toxicity of $CCl_4$ for mice after partial hepatectomy*

Determination of the $LD_{50}$ of $CCl_4$ for mice at various times after two-thirds hepatectomy (Table I) shows only a slight reduction after 12 h. While the $LD_{50}$ is less than the 95% confidence limit of the $LD_{50}$ in normal mice, this is not regarded as important because of possible interactions with the narcotic effects of the anaesthetic.

*Microsomal DMN-demethylase activity in mice after partial hepatectomy*

After two-thirds hepatectomy, DMN-demethylase activity in the remaining liver declined (Table III). The decline began between the third and sixth hours post-operatively when the values were 17·5 ± 2·5 and 11·2 ± 2·4 nmol HCHO/mg protein/30 min respectively, compared with a resting value of 16·7±3·3. Enzyme activity was back to normal between the sixth (144 h) and twelfth (288 h) days post-operatively, when the values were 12·5±4·0 and 16·9 ± 5·5 nmol HCHO/mg protein/30 min. Attention should be drawn to the greater scatter of these values at the later times.

*Liver microsomal DMN-demethylase activity in rats after partial hepatectomy*

In rats in which there is normally a lower level of liver DMN-demethylase activity there was a more immediate decline in activity after two-thirds hepatectomy (Table III) than in mice. The decline began within the first 3 h when the activity dropped from 12·3±2·9 to 8·0± 2·8 nmol HCHO/mg protein/30 min. As in mice, enzyme activity was back to normal between the sixth (144 h) and twelfth (288 h) days when the values were 9·6 ± 5·3 and 12·0 ± 5·7 nmol HCHO/mg protein/30 min respectively. Also, as in mice, attention should be drawn to the greater variability of the values at the later times.

*Histological aspects of toxicity of DMN and $CCl_4$ after partial hepatectomy in mice*

Characteristic centrilobular confluent coagulative necrosis in intact mice of this strain occurs only after a large dose of DMN, 11 mg/kg or more ($LD_{50}$ 11·4 mg/kg). The usual lesion seen after 24 h at this dose level consists of a large number of single dead cells scattered about the

FIG. 1.—Section of liver from mouse given 5 mg DMN/kg 24 h after two-thirds hepatectomy and killed 24 h later. The extent of necrosis is similar to that after 11 mg/kg in normal mice. At this stage after partial hepatectomy, mitosis is frequent in the absence of DMN treatment, but none is seen in this material. H. and E. ×75.

FIG. 2.—Section of liver from mouse given 11 mg DMN/kg 4 days after two-thirds hepatectomy and killed 24 h later. The lesion is typical of that seen after this dose of DMN in normal mice. H. and E. ×75.

central veins. The dead cells disappear rapidly by dissolution, fragmentation or phagocytosis by macrophages and adjacent liver cells, and later this leads to the characteristic blood lakes which, however, are less prominent than in the rat. Some confluent necrosis may be seen. At smaller doses only single necrotic cells are seen scattered in the central zones, but the number of these diminishes rapidly with the dose and they almost disappear at doses of 5 mg/kg.

In animals given 5 mg/kg either 6 h or 24 h after partial hepatectomy and killed 24 h later, considerable numbers of single necrotic cells and some confluent coagulative necrosis are present so that the lesion resembles that seen 24 h after 11 mg DMN/kg in intact mice (Fig. 1). When a dose of 5 mg/kg was given 4 days after two-thirds hepatectomy no significant lesion was seen in the liver but a dose of 11 mg/kg led to a lesion identical in character and extent of involvement to that which occurred in intact mice (Fig. 2). The other significant finding was that DMN (5 mg/kg) reduced the number of cells in mitosis usually seen from 36 h after partial hepatectomy (Fig. 2), and presumably the restoration of the liver mass is delayed.

The centrilobular necrosis produced by DMN in rats is histologically better known, but is produced by a dose of about

0·6 $LD_{50}$. Rats given about one-half this dose 12 h after partial hepatectomy show necrosis of similar extent. When dosed 48 h after partial hepatectomy the histological responses do not differ from those in intact rats.

The administration of $CCl_4$ 6 or 48 h after partial hepatectomy produced characteristic centrilobular coagulative necrosis of an extent that was not significantly different from that in normal mice given the same dose.

*Liver and kidney tumour formation in rats given DMN after partial hepatectomy*

The surviving animals injected with 10 mg DMN/kg at various times after one-third and two-thirds hepatectomy (Table II), as well as the control animals (untreated and sham hepatectomized) given 20 mg DMN/kg were maintained for 18 months, when all remaining animals were killed. A few animals that died in the first 9 months were ignored. Animals dying after 9 months were examined post mortem. The livers and kidneys were examined macroscopically for the presence of tumours which were characteristic nodules from 0·5 to 3·0 cm diameter. Livers often had a fine granular surface resembling a fine cirrhosis but the organs were not fibrotic. Kidneys otherwise appeared normal. Tumours were classified histologically on the same criteria as before (Pound *et al.*, 1973*b*), liver tumours as hepatocellular tumours or cholangiomata, kidney tumours as adenocarcinomata or mesenchymal tumours (Riopelle and Jasmin, 1969). The results are set out in Table IV.

The number of tumours in each experimental situation is too small to permit a detailed statistical analysis of the effect of the time interval between hepatectomy and the administration of DMN. Appropriate groups have been combined to formulate the statistics in Table IV. The yields of both hepatocellular tumours and of kidney tumours (all types combined) in the hepatectomized groups are greater than in the control group (which had twice the dose of DMN). The increase is significantly greater in two-thirds hepatectomized animals than in one-third hepatectomized animals. The yield of hepatocellular tumours in the period 24, 48 and 72 h after hepatectomy is greater than in the period 1, 6 and 12 h in the combined one-third and two-thirds hepatectomized groups; but in the one-third and two-thirds hepatectomized

TABLE IV.—*Number of Tumours of Liver and Kidneys of Rats given DMN*, 10 *mg/kg*, *at Different Times after Partial Hepatectomy, and in Control Animals, which had No Liver Removed, given DMN*, 20 *mg/kg*

| Extent of hepatectomy | | Control | Time after partial hepatectomy (h) 1 | 6 | 12 | 24 | 48 | 72 | Total numbers of tumours* |
|---|---|---|---|---|---|---|---|---|---|
| One-third removed | Animals | 36 (3) | 11 (4) | 9 (2) | 15 (1) | 12 (2) | 17 (2) | 19 (1) | 83 (12) |
| | H.C. | 1 | — | 1 | 2 | 4 | 3 | 5 | 15 |
| | Chol. | — | — | — | 1 | — | 1 | 1 | 3 |
| | Adeno. | — | 1 | 2 | 1 | 2 | 2 | 1 | 9 |
| | Mesen. | 2 | 2 | 1 | — | 2 | 3 | 2 | 10 |
| Two-thirds removed | Animals | | 6 (1) | 7 (1) | 8 (1) | 8 (2) | 15 (3) | 16 (3) | 60 (11) |
| | H.C. | | 2 | — | 2 | 5 | 8 | 6 | 23 |
| | Chol. | | — | 1 | 3 | 1 | — | 2 | 7 |
| | Adeno. | | 1 | 1 | 2 | 3 | 2 | 3 | 12 |
| | Mesen. | | 2 | 3 | 2 | 4 | 3 | 2 | 16 |

Number in parentheses is the number of animals that died before 9 months of age.
* = Excluding control animals, which had no liver removed.
H.C. = Hepatocellular tumours.
Chol. = Cholangiomata.
Adeno. = Adenocarcinomata of kidney.
Mesen. = Mesenchymal tumours of kidneys.

TABLE V.—*Statistical Data Relating to Results of Table IV*

| | | |
|---|---|---|
| Hepatocellular tumours | One-third hepatectomy > controls | $\chi^2 = 4\cdot37$, 1 d.f., $P < 0\cdot05$ |
| | Two-thirds hepatectomy > $\frac{1}{3}$ hepatectomy | $\chi^2 = 4\cdot54$, 1 d.f., $P < 0\cdot05$ |
| Hepatocellular tumours | * Period 24, 48, 72 h > period 1, 6, 12 h | $\chi^2 = 6\cdot02$, 1 d.f., $P < 0\cdot025$ |
| Kidney tumours | One-third hepatectomy > controls | $\chi^2 = 4\cdot30$, 1 d.f., $P < 0\cdot05$ |
| | Two-thirds hepatectomy > $\frac{1}{3}$ hepatectomy | $\chi^2 = 5\cdot01$, 1 d.f., $P < 0\cdot05$ |
| Kidney tumours | * Period 1, 6, 12 h *vs* period 24, 48, 72 h | $\chi^2 = 0\cdot02$, N.S. |

Yates correction for continuity has been applied.
* Results of one-third + two-thirds hepatectomized animals combined.

groups considered separately the results do not reach statistical significance ($\chi^2 = 2\cdot34$ and $\chi^2 = 3\cdot04$ respectively). On the other hand, there is no evidence of any such interval effect in the yield of kidney tumours. The yield of liver tumours in the periods 1, 6 and 12 h is greater after hepatectomy than in the controls but the increase is not statistically significant ($\chi^2 = 2\cdot5$, 1 d.f., $P > 0\cdot05$).

### DISCUSSION

The present experiments confirm that the yield of liver tumours produced' by DMN in rats is greater after partial hepatectomy (Craddock, 1971, 1973). The increase was greater after two-thirds than after one-third hepatectomy and was greater when DMN was given 24 h to 3 days after hepatectomy than in the first 12 h. These observations support the view that proliferating liver cells are more susceptible to this carcinogen (Craddock, 1971), as postulated for other carcinogens (Pound, 1968; Chernozemski and Warwick, 1970; Marquardt, Phillips and Bendich, 1972; Pound and Lawson, 1974*a*). In the rat liver DNA synthesis reached a peak 18–24 h after partial hepatectomy, mitotic activity a peak 6–12 h later, and the proliferative response was greater after two-thirds hepatectomy (Bucher and Malt, 1971).

A similar interpretation was advanced for the increased tumour yield in the liver of rats when DMN was given after a dose of carbon tetrachloride (Pound *et al.*, 1973*b*). In mice, a hepatonecrotic dose of $CCl_4$ given 48 h before DMN or diethylnitrosamine increased the number of hepatocellular tumours found after 12 months but the yield of kidney tumours was too small to examine statistically (Pound, unpublished data). It appears likely that the two species do not differ significantly in the issue involved and that mouse liver may also be more susceptible when proliferating rapidly.

Significant metabolic changes occurred in the liver after partial hepatectomy, not all of which are directly related to the proliferation of cells (Barker, Arcasoy and Smuckler, 1969; Bucher and Malt, 1971). In particular, the levels of activity of cytochrome P450 and many enzymes of the endoplasmic reticulum involved in drug metabolism were rapidly depressed for periods of 3–5 days and only slowly returned to normal (Barker *et al.*, 1969; Henderson and Kersten, 1970, 1971). A similar result is now reported in the depression of DMN-demethylase activity in liver microsome preparations from both rats and mice after partial hepatectomy. Metabolism of DMN to an active intermediate is a key step in the mechanism of its carcinogenic and hepatotoxic actions.

A single small dose of $CCl_4$ also depressed the level of activity of DMN-demethylase in liver microsomal preparations from mice and rats (Pound *et al.*, 1973*a*; Pound and Lawson, 1974*a*, 1975*a*), as well as the levels of cytochrome P450 and other microsomal enzymes in rats (Smuckler, Arrhenius and Hultin, 1967). This depression correlated with substantial protection against the acute toxic effects of a second dose of $CCl_4$ or of a dose of DMN in both rats and mice (Pound and Lawson, 1975*b*). Histological assessment of this protection in terms of the liver damage produced is complicated by the necrosis produced by the $CCl_4$ itself, which involves the susceptible centrilobular zones of the hepatic lobules.

However, after hepatectomy, when the susceptible centrilobular zones are retained in the remaining liver, the toxicity of DMN in mice, as measured by the $LD_{50}$ and the histological evidence of centrilobular necrosis, was increased even though the activity of microsomal enzymes was decreased. Craddock (1971, 1973), who was able to give only a much reduced dose of DMN to rats after hepatectomy, also found that the rate of metabolism of DMN *in vivo* was slowed in hepatectomized rats, as would perhaps be expected from the decreased activity of microsomal enzymes and the depleted amount of liver tissue. The increased toxicity may be contributed to by the small amount of remaining liver, which includes the susceptible central zones, but in any event the duration of exposure of the cells of all tissues to DMN was much increased when it was given up to about 5 days after hepatectomy.

Prolonged exposure of the cells to DMN probably explains the increased tumour yield in the kidneys, as a " dose effect ", similar to the case when a hepatocellular necrotic dose of $CCl_4$ increased the tumour yields in the kidneys of rats given DMN some time later (Pound *et al.*, 1973*b*), and to the increased yield of kidney tumours in rats in which microsomal enzyme activity in the liver was depressed by a low protein diet (McLean and Magee, 1970).

It is obvious that a similar " dose effect " must be brought to bear on the liver after hepatectomy. It would seem possible that the liver cells may be less susceptible to the hepatotoxic action of DMN than before partial hepatectomy were they subjected to the same load of DMN, but that the increased hepatocellular necrosis observed is the result of prolonged exposure of a reduced amount of liver tissue. This is usually largely replaced in the period 2–6 days.

It seems likely that the increased duration of exposure will explain the increased tumour yield when DMN is given up to 24 h after hepatectomy. When given after this time it seems possible that the further increase is determined by the proliferative state of the liver cells at the time, but clearly more information is needed to define precisely the relative roles of the various phenomena. It is to be noted that DMN decreased the number of cells in mitosis probably by interfering with DNA synthesis (Stewart and Magee, 1971), which still further obscures precise correlation with the susceptible phase of the cell cycle in the regenerating liver.

If, as seems probable, the stage in the cell cycle is a significant factor for the action of a carcinogen, then numerous considerations arise. In the first place if DMN-demethylase is, or is an index of, an enzyme involved in the activation of the carcinogen, the biochemical disturbances during cell proliferation will influence the production of tumours. Similarly, the action of any drug that affects the activity of the enzymes concerned will change the pattern of neoplastic development. Either the extent of the interaction of the carcinogen with cellular macromolecules may be changed or the pattern of interaction may be altered. For example, the altered conditions might favour an $O^6$-methylation (O'Connor, Capps and Craig, 1973) of a purine base or the formation of a triester phosphate (O'Connor, Margison and Craig, 1975) in the DNA chain. Alternately, directly or indirectly, the metabolic excision of the alkylated bases that occurs (O'Connor *et al.*, 1973) may be changed; or again the altered metabolic state of the cells may influence the necessary replicative step that results in the neoplastic manner of growth.

This work was supported by grants from the Queensland Cancer Fund and the Mayne Bequest Fund of the University of Queensland.

### REFERENCES

BAKER, R. C., COONS, L. B., & HODGSON, E. (1973) Low Speed Preparation of Microsomes: A Comparative Study. *Chem. Biol. Interactions*, **6**, 307.

BARKER, E. A., ARCASOY, M. & SMUCKLER, E. A. (1969) A Comparison of the Effects of Partial Surgical and Partial Chemical ($CCl_4$) Hepatectomy on Microsomal Cytochrome $b_5$ and $P_{450}$ and Oxidative N-Demethylation. *Agents and Actions*, **1**, 27.

BUCHER, N. L. R. & MALT, R. A. (1971) *Regeneration of Liver and Kidney*. Boston: Little, Brown and Company.

CHERNOZEMSKI, I. N. & WARWICK, G. P. (1970) Liver Regeneration and Induction of Hepatomas in $B6AF_1$ Mice by Urethan. *Cancer Res.*, **30**, 2685.

CRADDOCK, V. M. (1971) Liver Carcinomas Induced in Rats by Single Administration of Dimethylnitrosamine after Partial Hepatectomy. *J. natn. Cancer Inst.*, **47**, 899.

CRADDOCK, V. M. (1973) Induction of Liver Tumours in Rats by a Single Treatment with Nitroso Compounds given after Partial Hepatectomy. *Nature, Lond.*, **245**, 386.

CRADDOCK, V. M. & FREI, J. V. (1974) Induction of Liver Cell Adenomata in the Rat by a Single Treatment with N-methyl-nitrosourea given at Various Times after Partial Hepatectomy. *Br. J. Cancer*, **30**, 503.

DAVIES, D. J., GIGON, G. L. & GILLETTE, J. R. (1968) Sex Differences in the Kinetic Constants of N-dimethylation of Ethylmorphine by Rat Liver Microsomes. *Biochem. Pharmac.*, **17**, 1865.

GOA, J. (1953) Microbiuret Method for Protein Determination: Determination of Total Protein in Cerebro-spinal Fluid. *Scand. J. clin. Invest.*, **5**, 218.

GRÜNTHAL, D., HELLENBROICH, D. O., SÄNGER, P. & MAAS, H. (1970) Der Einfluss von partiellen Hepatektomien auf die Hepatomrate nach Diathylnitrosamin-Gaben. *Z. Naturforsch.*, **25**, 1277.

HENDERSON, P. T. & KERSTEN, K. J. (1970) Metabolism of Drugs during Rat Liver Regeneration. *Biochem. Pharmac.*, **19**, 2343.

HENDERSON, P. T. & KERSTEN, K. J. (1971) Alteration of Drug Metabolism during Rat Liver Regeneration. *Archs int. Pharmacodyn. Thér.*, **189**, 373.

HIGGINS, G. M. & ANDERSON, R. M. (1931) Experimental Pathology of the Liver. I. Restoration of the Liver of the White Rat Following Partial Surgical Removal. *Archs Path.*, **12**, 186.

MCLEAN, A. E. M. & MAGEE, P. N. (1970) Increased Renal Carcinogenesis by Dimethyl Nitrosamine in Protein Deficient Rats. *Br. J. exp. Path.*, **51**, 587.

MARQUARDT, H., PHILLIPS, F. S. & BENDICH, A. (1972) DNA Binding and Inhibition of DNA Synthesis after 7,12-Dimethylbenz(a)anthracene Administered During the Early Replicative Phase in Regenerating Rat Liver. *Cancer Res.*, **32**, 1810.

MILLER, J. A. & MILLER, E. C. (1969) The Metabolic Activation of Carcinogenic Aromatic Amines and Amides. *Prog. exp. Tumor Res.*, **11**, 273.

NASH, T. (1953) The Colorimetric Estimation of Formaldehyde by Means of the Hantzsche Reaction. *Biochem. J.*, **55**, 416.

O'CONNOR, P. J., CAPPS, M. J. & CRAIG, A. W. (1973) Comparative Studies of the Hepatocarcinogen N,N-Dimethylnitrosamine *in vivo*: Reaction Sites in Rat Liver DNA and the Significance of their Relative Stabilities. *Br. J. Cancer*, **27**, 153.

O'CONNOR, P. J., MARGISON, G. P. & CRAIG, A. W. (1975) Phosphotriesters in Rat Liver Deoxyribonucleic Acid after the Administration of the Carcinogen NN-Dimethylnitrosamine *in vivo*. *Biochem. J.*, **145**, 475.

POUND, A. W. (1968) Carcinogenesis and Cell Proliferation. *N.Z. med. J.* (*Special Issue*), **67**, 88.

POUND, A. W. (1972) Tumour Formation in Mice by Urethane Administered with Related Carbamates. *Br. J. Cancer*, **26**, 216.

POUND, A. W., HORN, L. & LAWSON, T. A. (1973*a*) Decreased Toxicity of Dimethylnitrosamine in Rats after Treatment with Carbon Tetrachloride. *Pathology*, **5**, 233.

POUND, A. W., LAWSON, T. A. & HORN, L. (1973*b*) Increased Carcinogenic Action of Dimethylnitrosamine after Prior Administration of Carbon Tetrachloride. *Br. J. Cancer*, **27**, 451.

POUND, A. W. & LAWSON, T. A. (1974*a*) Effects of Partial Hepatectomy on Carcinogenicity, Metabolism, and Binding to DNA of Ethyl Carbamate. *J. natn. Cancer Inst.*, **53**, 423.

POUND, A. W. & LAWSON, T. A. (1974*b*) Protection by a Small Dose of Carbon Tetrachloride against the Toxic Effects of Dimethylnitrosamine in Rats. *Br. J. exp. Path.*, **55**, 203.

POUND, A. W. & LAWSON, T. A. (1975*a*) Protection by Carbon Tetrachloride against the Toxic Effects of Dimethylnitrosamine in Mice. *Br. J. exp. Path.*, **56**, 77.

POUND, A. W. & LAWSON, T. A. (1975*b*) Reduction of Carbon Tetrachloride Toxicity by Prior Administration of a Single Small Dose in Mice and Rats. *Br. J. exp. Path.*, **56**, 172.

RIOPELLE, J. L. & JASMIN, G. (1969) Nature, Classification, and Nomenclature of Kidney Tumors Induced in the Rat by Dimethylnitrosamine. *J. natn. Cancer Inst.*, **42**, 643.

SMUCKLER, E. A., ARRHENIUS, E. & HULTIN, T. (1967) Alterations in Microsomal Electron Transport, Oxidative N-Demethylation and Azo-Dye Cleavage in Carbon Tetrachloride and Dimethylnitrosamine-induced Liver Injury. *Biochem. J.*, **103**, 55.

STEWART, B. W. & MAGEE, P. N. (1971) Effect of a Single Dose of Dimethylnitrosamine on Biosynthesis of Nucleic Acid and Protein in Rat Liver and Kidney. *Biochem. J.*, **125**, 943.

VENKATESAN, N., ARCOS, M. F. & ARGUS, J. C. (1970) Mechanism of 3-Methylcholanthracene-induced Inhibition of Dimethylnitrosamine Demethylase in the Rat Liver. *Cancer Res.*, **30**, 2556.

WEIL, C. S. (1952) Tables for Convenient Calculation of Median-effective Dose ($LD_{50}$ or $ED_{50}$) and Instructions in their Use. *Biometrics*, **8**, 249.